# Extraction optimization, structure features, and bioactivities of two polysaccharides from *Corydalis decumbens*

**DOI:** 10.1371/journal.pone.0284413

**Published:** 2023-04-13

**Authors:** Zhaojing Wang, Dianhui Luo

**Affiliations:** Department of Bioengineering and Biotechnology, Fujian Provincial Key Laboratory of Biochemical Technology, Huaqiao University, Xiamen, Fujian, People’s Republic of China; USDA-ARS Southeast Area, UNITED STATES

## Abstract

Two polysaccharides (CPS1 and CPW2) from *Corydalis decumbens* were obtained to develop insights into natural medical resources. Optimal extraction conditions of total sugars were researched using the method of response surface methodology, polysaccharides were purified using a combination of ethanol precipitation and anion-exchange chromatography, and structure features were analyzed by scanning electron microscopy, transmission electron microscopy, and Congo-red assay. The bioactivities were estimated in terms of antioxidant and anti-inflammatory effects. Total sugars were extracted with an experimental yield of 32.74% under optimum conditions. CPS1 and CPW2 were purified with yields of 12.01% and 8.23%, respectively. CPS1 was a unique polysaccharide with a molecular weight (Mw) of 360 kDa and consisted of glucose, galactose, mannose, and arabinose in a ratio of 4.9:2.0:1:1.9, and CPW2 was composed of glucose with the Mw of 550 kDa. CPS1 possessed a four-helix conformation, and CPW2 was identified as a linear molecule without branched and entangled chains. The mRNA expressions of TNF-α (71.80%), IL-1β (56.55%), IL-6 (43.98%), and COX-2 (91.88%) in LPS-stimulated RAW 264.7 cells were significantly inhibited by 75 μg/mL CPS1 (*P* < 0.0001), while CPW2 showed lower inhibitory effects than CPS1. Compared with CPW2, CPS1 showed stronger scavenging abilities for hydroxyl (EC_50_ = 520.46 μg/mL), ABTS (EC_50_ = 533.99 μg/mL), and superoxide (EC_50_ = 1512.06 μg/mL) radicals. CPS1 with four-helix conformation exhibited more outstanding bioactivities than CPW2 without entangled chains.

## Introduction

*Corydalis decumbens* is an herb belonging to Papaveraceae families and the species are distributed in different provinces in China [1]. Its dried stem tubers are bitter and pungent and have been utilized as a traditional Chinese medicine for thousands of years [2]. Modern pharmacological studies have shown that *C*. *decumbens* has anti-inflammatory, anti-cerebral infarction, anti-arrhythmia, and brain nerve protection effects [3,4]. It has been reported that *C*. *decumbens* comprises mainly alkaloids, such as protopine, tetrahydropalmatine and palmatine, and then its health-beneficial effects are also owing to the alkaloid ingredients [5]. Polysaccharides, as important activity substances, exist broadly in the water extract of plants and may play a marked role in beneficial ingredients [6]. However, there is no report concerning chemical compositions, structural characteristics, anti-inflammatory effects, and antioxidant activities of polysaccharides from *C*. *decumbens*.

The over-expression of inflammatory factors can cause multiple diseases, including intracerebral hemorrhage, Alzheimer’s disease, and atherosclerosis [7,8]. Some toxic substances, such as chemokines and inflammatory factors, will be released and aggravate the damage of neurons after the onset of intracerebral hemorrhage [9]. It has been reported that the chemokine TNF-α can increase plaque deposition, lead to neurotoxicity and increase significantly in Alzheimer’s patients [10]. So, it is necessary to control the over-expression of inflammatory factors. Some studies have shown that natural compounds from extracts of medicinal plants have good anti-inflammatory effects attributed to their polysaccharide compounds [11]. For example, Wang et al. [12] reported that the polysaccharide extracted from *Gynostemma pentaphyllum* herb showed a high anti-inflammatory activity by decreasing the levels of the factors TNF-α and IL-6. As much, polysaccharides from medicinal plants can also show excellent antioxidant capacities. Gu et al. [6] found that three polysaccharides from *Sagittaria sagittifolia* L. exhibited stronger antioxidant activities by scavenging reactive oxygen species (ROS) including ABTS, DPPH, and hydroxyl radicals. Neurons are most susceptible to oxidative injury by ROS and oxidative stress is also a feature of neurological diseases [13]. Therefore, it remains to be one of the interesting research fields to search for natural anti-inflammatory and antioxidant polysaccharide compounds.

In the present study, we isolated two novel polysaccharides from *C*. *decumbens*, demonstrated fine structure features, and related it to biological functions such as anti-inflammatory and antioxidant activities. To our knowledge, this is the first time that detailed properties of polysaccharides from *C*. *decumbens* were reported. This study may also provide new insights into beneficial ingredients from *C*. *decumbens* and facilitate its pharmaceutical application, especially as potential preventive-therapeutic agents for the treatment of inflammation-related chronic human diseases such as intracerebral hemorrhage and atherosclerosis.

## Materials and methods

### Materials and chemicals

Dried stem tubers from *C*. *decumbens* were purchased in a local drugstore and their origin was in Jiangxi Province, China. The species was identified as *C*. *decumbens* by professor Zhihong Huang, Huaqiao University, China.

RAW 264.7 cells were obtained from ATCC. Most of the chemicals, including DEAE Sepharose CL-6B (GE-Healthcare, USA), fetal bovine serum (FBS, Hyclone, Logan, UT), lipopolysaccharide (LPS, Sigma, St. Louis, MO), Total RNA Super Extraction Kit (LS1040, Promega, Madison, WI, US), Reverse Transcription System (A5001, Promega, Madison, WI, US), qPCR Master Mix (A6001, Promega, Madison, WI, US) were provided by a local reagent agency (Xiamen Tagene Biotechnology Co., Ltd.), and then they were of analytical grade.

### Extraction optimization of total sugars

#### Extraction of total sugars

Dry *C*. *decumbens* powder (1 g) was added to a certain volume of distilled water at a selected temperature for some time in each test. After the water extraction, the supernatant was collected to determine total sugar content using the phenol-sulfuric acid method, and the yield of total sugars (%, w/w) could be obtained [14].

#### Experimental design and statistical analysis

The single-factor investigation was conducted following the procedure [14] described as early, and then a Box–Behnken design (BBD) with three-level-three-factor was used to optimize the extraction process of total sugars according to the single-factor results. The independent variables included liquid-to-solid ratio (A), extraction time (B), and extraction temperature (C), while response values were total sugar yields (Y). 17 experiments were carried out, and data were analyzed using the Design-Expert software (Version 11, Stat-Ease Inc., Minneapolis, MN, USA).

#### Extraction and purification tests of polysaccharides

50 g of dried stem tubers from *C*. *decumbens* were added into 3 L distilled water and incubated at a 68 ˚C water bath for 250 min to obtain extracting solution. The solution was concentrated to 100 mL after filtrating against insoluble residue and 2 volumes of ethanol were added for 15 h at 4 ˚C. After centrifugation, the precipitate was freeze-dried to obtain a crude product (named CP1) and the supernatant was added in 200 mL ethanol for another 15 h at 4 ˚C, followed by centrifugation to recover another crude product (named as CP2) from the solution.

300 mg of CP1 was dissolved into 15 mL distilled water and the solution was separated by a DEAE-Sepharose CL-6B column (4.6 cm × 37 cm) with distilled water as the eluent for 21 h at a flow rate of 1 mL/min. After that, the elute was replaced with 600 mL of 0.25 and 0.45 mol/L NaCl solutions for the linear gradient elution at the same flow rate. The collecting solution was combined from 370 to 500 mL to obtain a purified *C*. *decumbens* polysaccharide (CPS1) after dialysis against distilled water for 36 h (5000 Da Mw cut off), concentrating, and freeze-drying.

300 mg of CP2 was dissolved with 15 mL distilled water and purified using the DEAE-Sepharose CL-6B column. The eluent was distilled water at a flow rate of 0.8 mL/min, and then the collecting solution (280–569 mL) was freeze-dried after dialysis to obtain another purified *C*. *decumbens* polysaccharide (CPW2).

#### High-performance size-exclusion chromatography (HPSEC) analysis

The homogeneity of polysaccharides was determined by injecting a 0.5 mg/mL sample after the filtration with a 0.22 μm nylon membrane into an 1100 system (Agilent Technologies, Palo Alto, CA, USA) equipped with a refractive index detector (RID) and a Shodex Sugar KS-804 column (Showa Denko K.K, Japan), and eluting with ultrapure water under 50 ˚C column temperature at a flow rate of 1 mL/min [15].

Based on the retention times of standard dextran (T10, T40, T70, and T500) in HPSEC by RID, the molecular weight (Mw) distribution of polysaccharides was able to be determined [15].

#### UV spectrum

To measure contaminates such as protein and nucleic acid, 1 mg/mL of sample water solution was recorded on a UV-1800PC spectrophotometer at the absorbance mode from 190 to 800 nm.

#### Chemical analysis

To measure the content of chemical constituents, the phenol-sulfuric acid method [16] was used for the determination of total glucose content, and the sulfuric acid carbazole [17] and Bradford’s methods [18] were used to determine the content of uronic acid and protein, respectively.

#### Fourier transform infrared spectroscopy (FTIR) analysis

For the identification of specifical functional groups in polysaccharide molecules, the FTIR method was used, and the spectra were recorded on a Fourier Transform Infrared Spectrometer (Nicolet iS10, Thermo Fisher Scientific, Waltham, MA, USA) at the absorbance mode from 4000 to 500 cm^-1^.

#### Monosaccharide composition test

The monosaccharide compositions of polysaccharides were determined using the method described earlier [19]. Briefly, the polysaccharide sample was hydrolyzed with 1 mol/L H_2_SO_4_ for 8 h at 100 ˚C, followed by neutralizing with barium carbonate to pH 7.0 and freeze-drying. After the dried products were derived using trimethylsilylation reagents, derivative compounds were identified on a gas chromatography (GC) system (7890, Agilent Technologies, Palo Alto, CA, USA) fitted with an HP-5 column (30 m × 0.25 mm × 0.25 μm). The detector was a flame-ionization detector (FID) and the column was run under the condition of programmed temperatures (160–180 ˚C at 20 ˚C/min, 180–220 ˚C at 8 ˚C/min, hold 2 min).

#### Congo red assay

Congo red assay was used to determine the helix conformation of polysaccharides. 80 μmol/L Congo red solution was mixed with 0.5 mg/mL sample solution in equal volumes, followed by adding 4 mol/L NaOH to adjust the mixture solutions to different final concentrations (0.1–0.5 mol/L) and standing for 10 min at room temperature. After that, UV spectra were recorded on a UV-1800PC spectrophotometer at a range of 400–700 nm, by which the maximum absorption wavelengths (λ_max_) of different solutions could be acquired [20].

#### Transmission electron microscopy (TEM) observation

TEM was used to observe the molecular morphology of polysaccharides. 1 mg/mL of polysaccharide solution (2 mL) was mixed with 1 mg/mL sodium dodecyl sulfate (SDS) solution (2 mL), and the mixed solution was incubated at an 80 ˚C water bath for 2 h and allowed to continue for another 2 h after diluting with distilled water to 5 μg/mL [21]. After a single droplet of reaction solution passing through a 0.22 μm cellulose membrane was deposited on a carbon film of 200 mesh and dried at room temperature, the specimen was visualized on TEM (H-7650, Hitachi High-Technologies Corporation, Tokyo, Japan).

#### Scanning electron microscopy (SEM) observation

The microstructure of polysaccharides was investigated by grounding sample powder onto a metal platform, sputtering with a thin layer of platinum, and using an SEM (S-4800, Hitachi High-Technologies Corporation, Tokyo, Japan) to observe at 5.0 kV accelerating voltage [22].

#### Analysis of anti-inflammatory property

*Cell culture and cytotoxicity tests*. RAW264.7 cells were cultured in a 5% CO_2_ atmosphere at 37°C for 48 h with high glucose DMEM medium containing 10% FBS and 1% penicillin. Using the MTT method, the cytotoxicity test of polysaccharides against RAW264.7 cells was carried out with mass concentrations of 600, 300, 150, 75, 25 and 1 μg/mL.

*Anti-inflammatory tests*. According to the results from the cytotoxicity tests, the polysaccharide samples (CPS1 and CPW2) with the amount of 150 and 75 μg/mL were used for the anti-inflammatory tests. All the anti-inflammatory tests included blank, negative control, positive control, and sample groups. After RAW264.7 cells were cultured on 6-well plates for 24 h, 75 μg/mL dexamethasone (DXMS), 150 μg/mL CPS1, 75 μg/mL CPS1, 150 μg/mL CPW2 and 75 μg/mL CPW2 were added into a positive control group and four sample groups, respectively [23]. The experiment groups were allowed to continue for another 20 h, followed by adding 1 μg/mL LPS into all the groups except the blank for 4 h to construct inflammatory models.

*Determination of mRNA expression levels*. The total RNA for each group was extracted by a total RNA isolation system with the manufacturer’s instruction, after that, the cDNA reverse transcription was carried out using a reverse transcription system under the experimental conditions of 42°C for 60 min and 70°C for 15 min. The target genes, including glyceraldehyde-3-phosphate dehydrogenase (GAPDH), TNF-α, IL-1β, IL-6, and COX-2, were amplified using a Real-Time PCR System (Roche 480II, Applied Biosystems). The primer sequences of target genes were provided in the previous report [24] and the experimental conditions were 95°C for 15 sec, 60°C for 30 sec, and 72°C for 30 sec (45 cycles) [23,25,26]. The molecular size of amplified genes was identified using the method of agarose gel electrophoresis. According to the C_T_ values and using the 2^-ΔΔCT^ method, the mRNA expression levels of the inflammatory factors relative to the GAPDH gene were able to be obtained.

#### Analysis of antioxidant property

For the identification of antioxidant activities, scavenging assays of superoxide [27], hydroxyl [28], and ABTS [6] radicals were conducted. The related steps were described as follows.

*Superoxide radical assay*. 0.1 mL sample solution of different reaction concentrations (15–3000 μg/mL) was mixed with 1 mL of 557 μmol/L NADH, 45 μmol/L PMS, and 108 μmol/L NBT solutions, followed by keeping at room temperature for 5 min and measuring the absorption values at 560 nm.

*Hydroxyl radical assay*. 0.1 mL of sample solution (10–4000 μg/mL reaction concentrations) was mixed in sequence with a mixture (0.6 mL, including 2.67 mmol/L deoxyribose and 0.13 mmol/L EDTA), 0.4 mmol/L ferrous ammonium sulfate (0.2 mL), 2.0 mmol/L ascorbic acid (0.05 mL) and 20 mmol/L H_2_O_2_ (0.05 mL), and incubated at a 37°C water bath for 15 min, followed by boiling for 15 min and cooling to room temperature after adding 1 mL of 1.0% thiobarbituric acid and 2.0% trichloroacetic acid. Then the absorbance of reaction solutions was measured at 532 nm.

*ABTS radical assay*. For the ABTS+ solution, 2.45 mmol/L persulfuric acid solution was dissolved into 7 mmol/L ABTS solution for 16 h in the dark, followed by diluting it 20 times with distilled water to obtain the working concentration. Sample solutions (1.25–5000 μg/mL reaction concentrations) were added to the ABTS+ working solution for 10 min in the dark and the absorbance was measured at 734 nm.

*Scavenging rate*. The scavenging rate for each radical was calculated based on the following formula.

Radical scavenging rate (%) = (1 ˗ (A_1_ ˗ A_2_) / A_0_) × 100%

Where A_1_ is the absorbance of the sample, A_0_ is the absorbance of the control (distilled water without sample), and A_2_ is the background absorbance.

*Statistical analysis*. All the experiments were repeated three times. Data were analyzed using the Origin Software 2017 software and determined as mean ± standard deviation (S.D.). Statistical significance differences were estimated using the method of one-way analysis for variance (ANOVA) and values of *P* < 0.05 were considered to be statistically significant.

## Results and discussion

### Extraction optimization of total sugars

**Fig 1:**
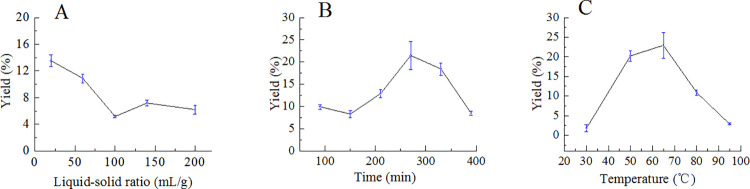
Effects of liquid-solid ratio (A), extraction time (B), and extraction temperature (C) on the yields of total sugars from *C*. *decumbens*.

Results from the single-factor investigation were demonstrated in Fig 1. The results showed that total sugar yields decreased with the increase of liquid-solid ratio from 20 to 200 (Fig 1A) and increased with the increase of extraction time from 90 to 270 min (Fig 1B). The yields were below 10% after the liquid-solid ratio reached 100 or when extraction time was below 150 min, while yields increased with elevating temperatures from 30 to 65 ˚C and decreased gradually after 65 ˚C (Fig 1C). In addition, the factor of extraction numbers was also investigated. The extraction numbers were selected from 1 to 5, and total yields were 22.92%, 25.45%, 25.11%, 24.87%, 23.23% in one condition (60 mL/g, 60 min, 65 ˚C), and 12.89%, 14.13%, 14.91%, 13.87%, 12.19% in another condition (60 mL/g, 210 min, 80 ˚C). The results suggested that the changes in total yields were unobvious with the increase of extraction numbers, and then the factor of extraction numbers will not be included in the next experiment considering the extraction cost.

According to the single-factor results, the Box–Behnken design was assigned and the results of 17 experiments were listed in Table 1. Using the Design-Expert Software, a regression model was obtained and described as follows.

Y = -166.55168+0.122058A+0.531025B+3.78316C+0.000450AB-0.000288AC-0.000475BC-0.001738A^2^-0.001046B^2^-0.026881C^2^。

**Table 1:** Box–Behnken experimental design and results for the yield of total sugar.

Run	Coded variable levels			Yield (%)
	A (mL/g)	B (min)	C (˚C)	
1	-1(10/1)	-1(150)	0(65)	20.144
2	1(100/1)	-1(150)	0(65)	14.103
3	-1(10/1)	1(330)	0(65)	22.163
4	1(100/1)	1(330)	0(65)	23.413
5	-1(10/1)	0(240)	-1(50)	15.409
6	1(100/1)	0(240)	-1(50)	21.827
7	-1(10/1)	0(240)	1(95)	23.317
8	1(100/1)	0(240)	1(95)	28.958
9	0(55/1)	-1(150)	-1(50)	14.488
10	0(55/1)	1(330)	-1(50)	18.366
11	0(55/1)	-1(150)	1(95)	17.771
12	0(55/1)	1(330)	1(95)	19.083
13	0(55/1)	0(240)	0(65)	30.321
14	0(55/1)	0(240)	0(65)	32.177
15	0(55/1)	0(240)	0(65)	33.427
16	0(55/1)	0(240)	0(65)	34.287
17	0(55/1)	0(240)	0(65)	29.516

Statistical analysis showed that a *p*-value was 0.0083 (*p* < 0.05), lack of fit was 0.0889 (*>* 0.05), and the R^2^ value for the model was 0.9021, indicating that the proposed model was suitable to calculate the extraction yield of total sugars from *C*. *decumbens*.

Using the model, optimum conditions and a predicted optimum response value were achieved. Under the predicted optimum conditions (A = 60 mL/g, B = 250 min, C = 68 ˚C), an experimental yield was (32.74 ± 0.072)%, which matched well with the expected value of 32.38%.

### Extraction and purification of polysaccharides

**Fig 2:**
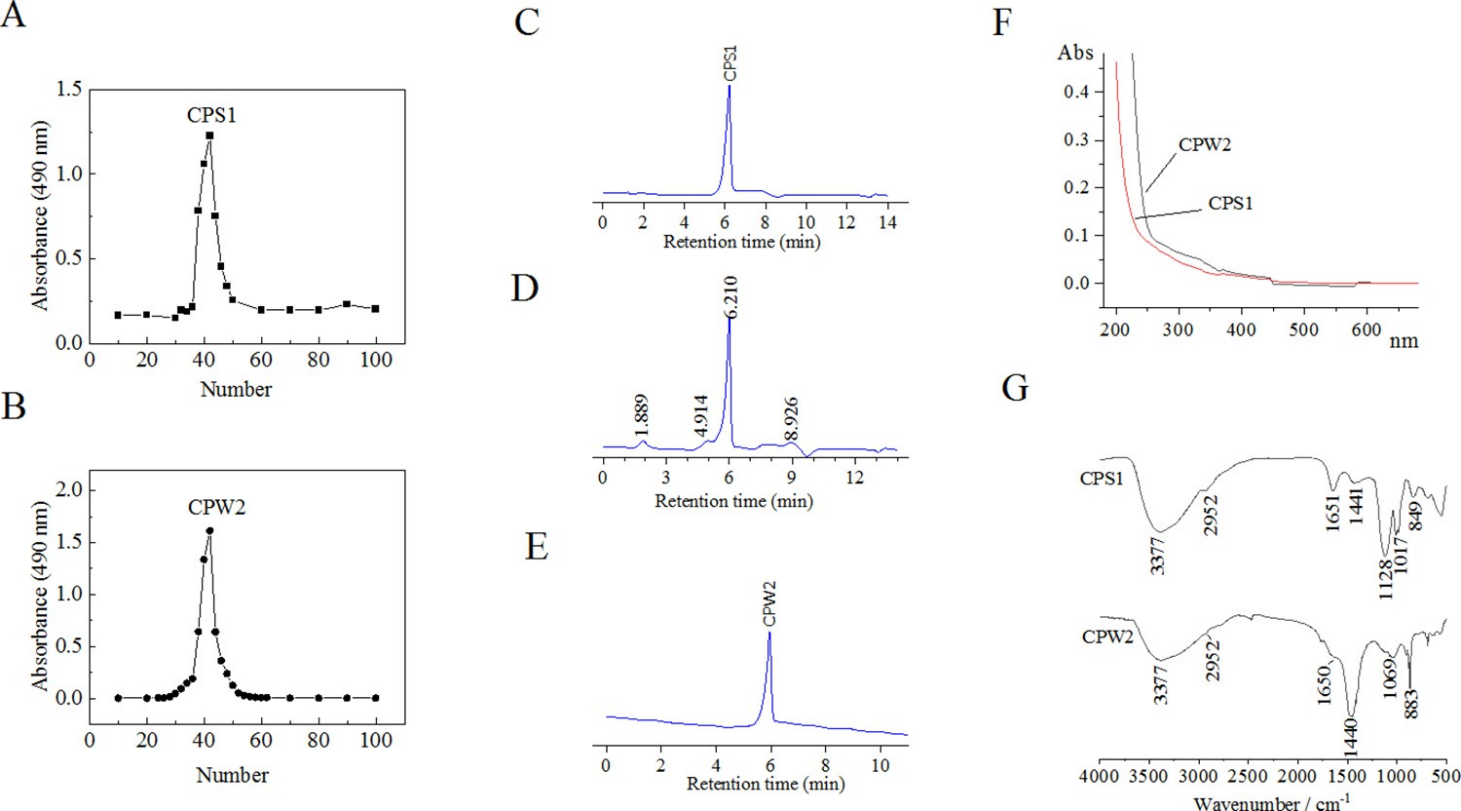
DEAE-Sepharose CL-6B elution profiles (A-B), HPSEC chromatograms (C-E), UV spectrum (F), and FTIR spectra (G) of CPS1 and CPW2.

Based on the hot-water extraction, crude products after ethanol precipitation were obtained from *C*. *decumbens*. Other high Mw compounds such as nucleic acid and protein were also obtainable under the hot-water extraction condition, which would create difficulties for the extraction of polysaccharides. To recover polysaccharides from the crude products, ethanol of different concentrations was used to separate two polysaccharide components (CP1 and CP2) into different grades with yields of 24.82% and 17.47%, respectively. By using the DEAE-Sepharose CL-6B (Fig 2A and 2B) combined with different elution conditions, two purified *C*. *decumbens* polysaccharides (CPS1 and CPW2) were further obtained from CP1 and CP2, respectively, which make both the yields to decrease to 12.01% and 8.23%.

### Homogeneity of CPS1 and CPW2

The HPSEC profiles of CPS1 and CPW2 in a Sugar KS-804 column by RID were demonstrated in Fig 2. For the CPS1 molecule, the elute was 0.24–0.45 mol/L NaCl solutions in the DEAE-Sepharose CL-6B chromatography, and a single peak at 6.21 min was observed in the HPSEC profile of CPS1 (Fig 2C). To study gradient elution conditions, a polysaccharide was initially achieved with different elution solutions (0–1 mol/L NaCl) in the purification process of DEAE-Sepharose CL-6B chromatography, and its HPSEC profile was presented in Fig 2D. As shown in Fig 2D, peaks at 1.89, 4.91 and 8.93 min were derived from other impurity substances in the polysaccharide by comparing with the HPSEC profile of CPS1 (Fig 2C, 6.21 min), while the impurity contents were calculated to be 35% according to their peak areas. These results indicated the special elution condition in the purification process of *C*. *decumbens* polysaccharide was able simply and effectively to help obtain the single and purified polysaccharide CPS1. The HPSEC profile of CPW2 was demonstrated in Fig 2E, in which a singular narrow peak at 5.95 min was observed, which indicated that CPW2 was also a single component. The UV spectra of CPS1 and CPW2 were shown in Fig 2F, where smooth curves were observed in CPS1 and CPW2 molecules, suggesting that containments covering protein and nucleic acid did not exist in both molecules.

### Molecular weight (Mw)

According to the data from standard dextran in HPSEC, a calibration curve of Mw was determined as y = -3.92x + 6.31 (R^2^ = 0.99). Based on the calibration curve and the retention times of CPS1 and CPW2 in HPSEC by RID, the Mw of CPS1 was calculated to be 360 kDa and that of CPW2 was 550 kDa.

### FTIR spectroscopic analysis

Using the FTIR, functional groups of compounds were identified, by which we could conclude the type of isolated compound. FTIR spectra of CPS1 and CPW2 were presented in Fig 2G, where the peaks at 3377, 2952, 1650–1651, 1440–1441, and 1017–1128 cm^-1^ were attributed from O-H, stretching C-H, C = O, angle C-H, and C-O-C bonds, respectively. All of the bonds were regarded to be the characteristic functional groups of carbohydrate compounds [29]. In addition, the peak at 849 cm^-1^ in CPS1 was assigned to the α glycosidic bond, and the peak at 883 cm^-1^ in CPW2 was derived from the β glycosidic bond, suggesting the existence of different configurations between CPS1 and CPW2.

### Molecular compositions

**Fig 3:**
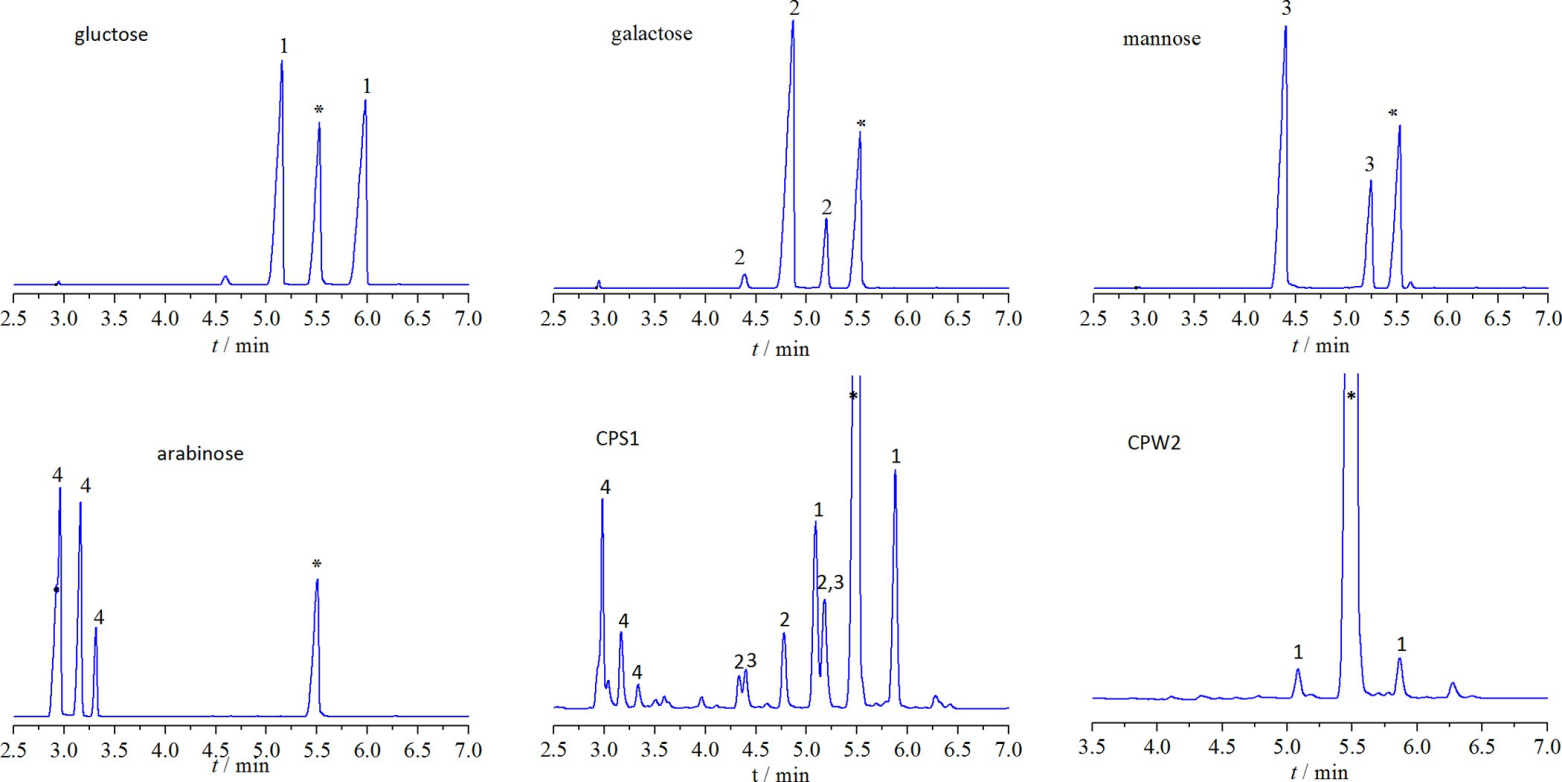
GC chromatograms of derivatives from monosaccharide standards, CPS1, and CPW2. * represents mannitol, 1–4 refer to derivatives from glucose, galactose, mannose, and arabinose.

Total carbohydrate contents in CPS1 and CPW2 were 98.36 ± 1.14% and 97.86 ± 2.02%, respectively. The dominant component was neutral sugar and there was no uronic acid in both of the polysaccharides. Using GC fitted with the HP-5 column, the derivatization products after the hydrolysis and derivatization of CPS1 and CPW2 were identified, and GC chromatograms were presented in Fig 3. Mannitol, as an internal standard substance, was added to the tests and marked to be an asterisk in Fig 3. By comparison of data with the GC chromatograms of standard monosaccharides, we could conclude that CPS1 was composed of glucose, galactose, mannose and arabinose in a molar ratio of 4.9:2.0:1:1.9 and CPW2 comprised of glucose.

### SEM analysis

**Fig 4:**
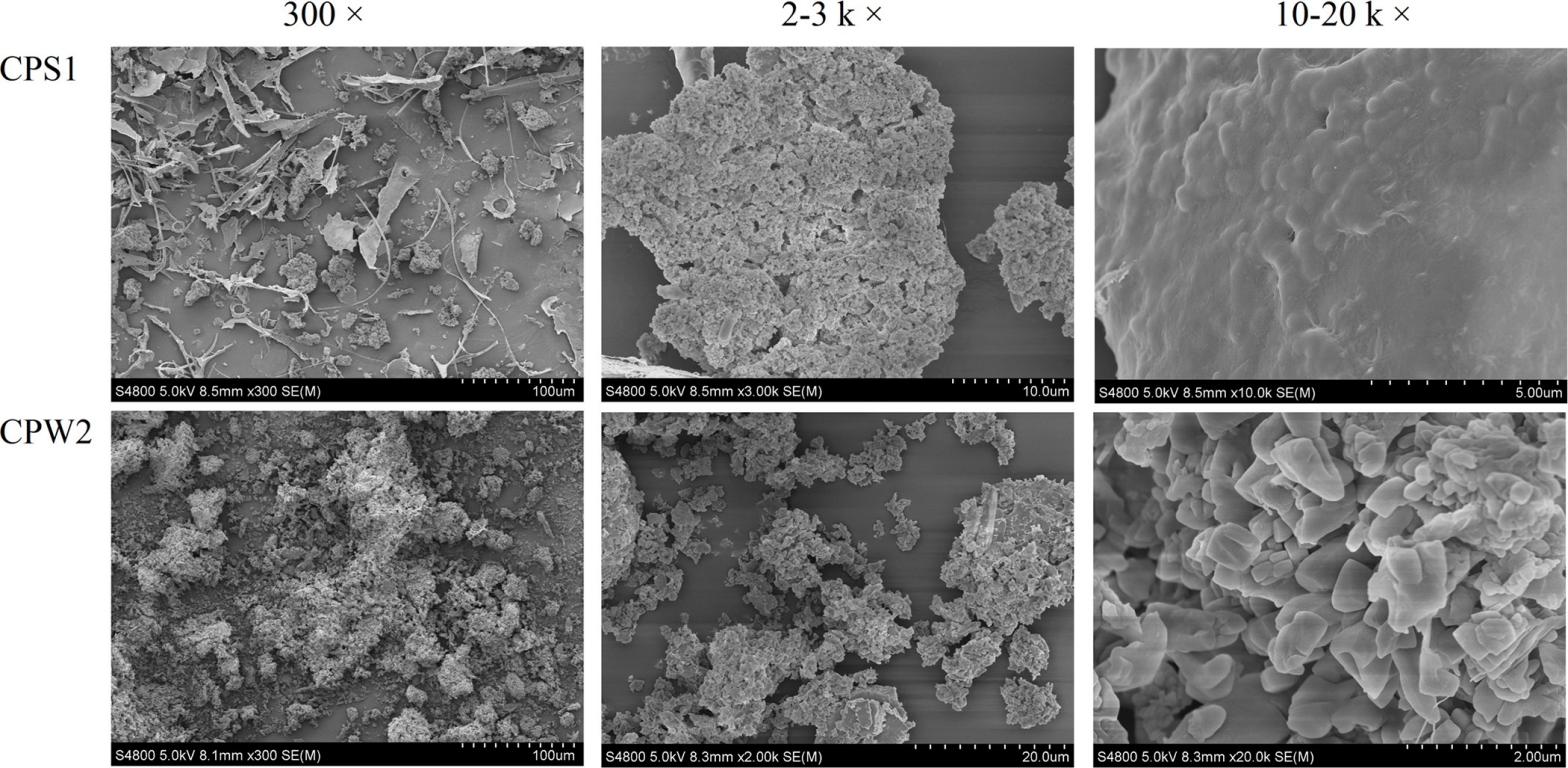
SEM images of CPS1 and CPW2 (300 × - 20 k ×).

The surface morphology of CPS1 and CPW2 was observed using SEM and the SEM images were presented in Fig 4 at magnifications of 300–20 k. As shown in Fig 4, the image of CPS1 at a magnification of 300 presented some irregular particles with non-uniform sizes. The rough surface with some holes was observed in the image with a magnification of 3000, which indicated the existence of a branched structure in the CPS1 molecule [22]. The image at a magnification of 10 k displayed many inhomogeneous lumps, and the agglomerations suggested that there were aggregated chains in polysaccharide CPS1 and the structure of CPS1 was entangled [20]. These deductions were further supported by the following results from TEM and Congo red tests.

The image of CPW2 at magnifications of 300 and 2000 presented some loose particles with a relatively flaky surface. At the magnification of 20 k, it could be observed that CPW2 was mainly composed of regular slices, and all the slices were stacked together. The schistose surface in CPW2 was even without holes based on the high magnification image, indicating that the CPW2 molecule was a linear structure without branched and entangled chains, which was further confirmed by the TEM results.

### TEM analysis

**Fig 5:**
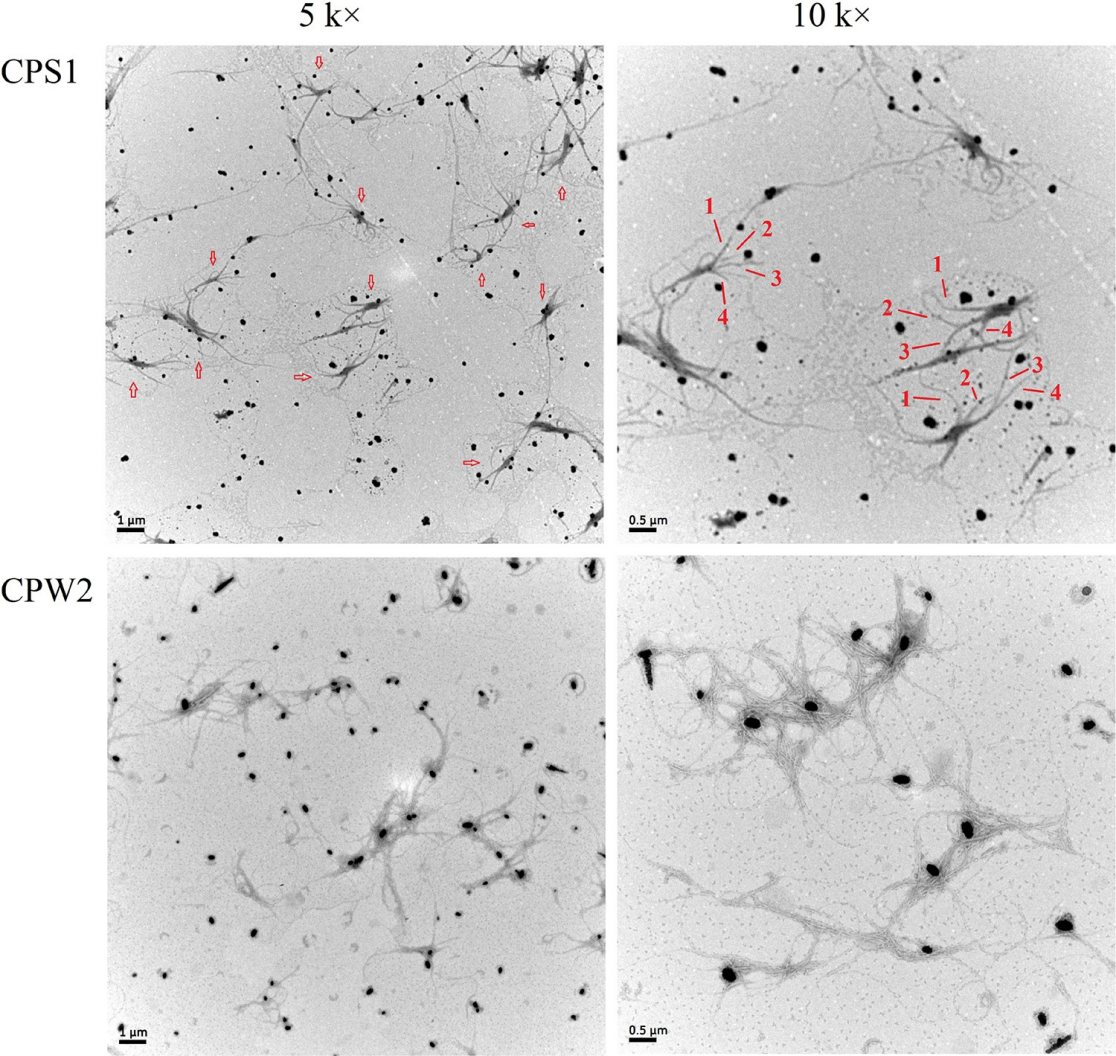
TEM images of CPS1 and CPW2 (5 k ×, 10 k ×).

TEM is a useful tool to identify the microscopic morphology, which is important to characterize the fine structure of polysaccharides. After polysaccharides are dispersed by the SDS solution, it is easy to observe the molecule morphology using TEM [30]. As shown in Fig 5, the TEM image of CPS1 at a magnification of 5000 displayed many subunits (labeled with arrows in Fig 5), and one subunit connected with another. When it had been magnified to 10 k, the molecule morphology with four single chains in CPS1 was visible and the chains trended to form an entangled structure. Similar to the triple-helix conformation, CPS1 molecules contained even more than three entangled chains. Side chains were also observed in CPS1 molecules, by which one subunit with four-helix chains combined with another. The results matched well with the above SEM deductions.

For the CPW2 molecule, TEM images showed that CPW2 possessed many single chains without entangled and hairy chains, which was similar to the results from SEM analysis. As described in previous references [6,31,32], the triple-helix structure for polysaccharide molecules is helpful to improve its biological activities such as antitumor, immunoregulation, and anti-inflammation activities, therefore the structural similarity of CPS1 with four-helix conformation to that indicated it could have similar outstanding bioactivities.

### Helix structure

Using the Congo red test, the triple-helix structure of polysaccharides can be simply evidenced [31]. After polysaccharides possessing helix conformation are mixed with Congo red solution, the special complex can be formed and the λ_max_ value of the complex will be higher than that of the Congo red control. However, the helix conformation will be damaged by strong alkaline (NaOH), and then the λ_max_ value of the Congo red-polysaccharide complex move to a low wavelength. To identify the four-helix conformation, Congo red tests were also applicable, which were described as followed.

**Fig 6:**
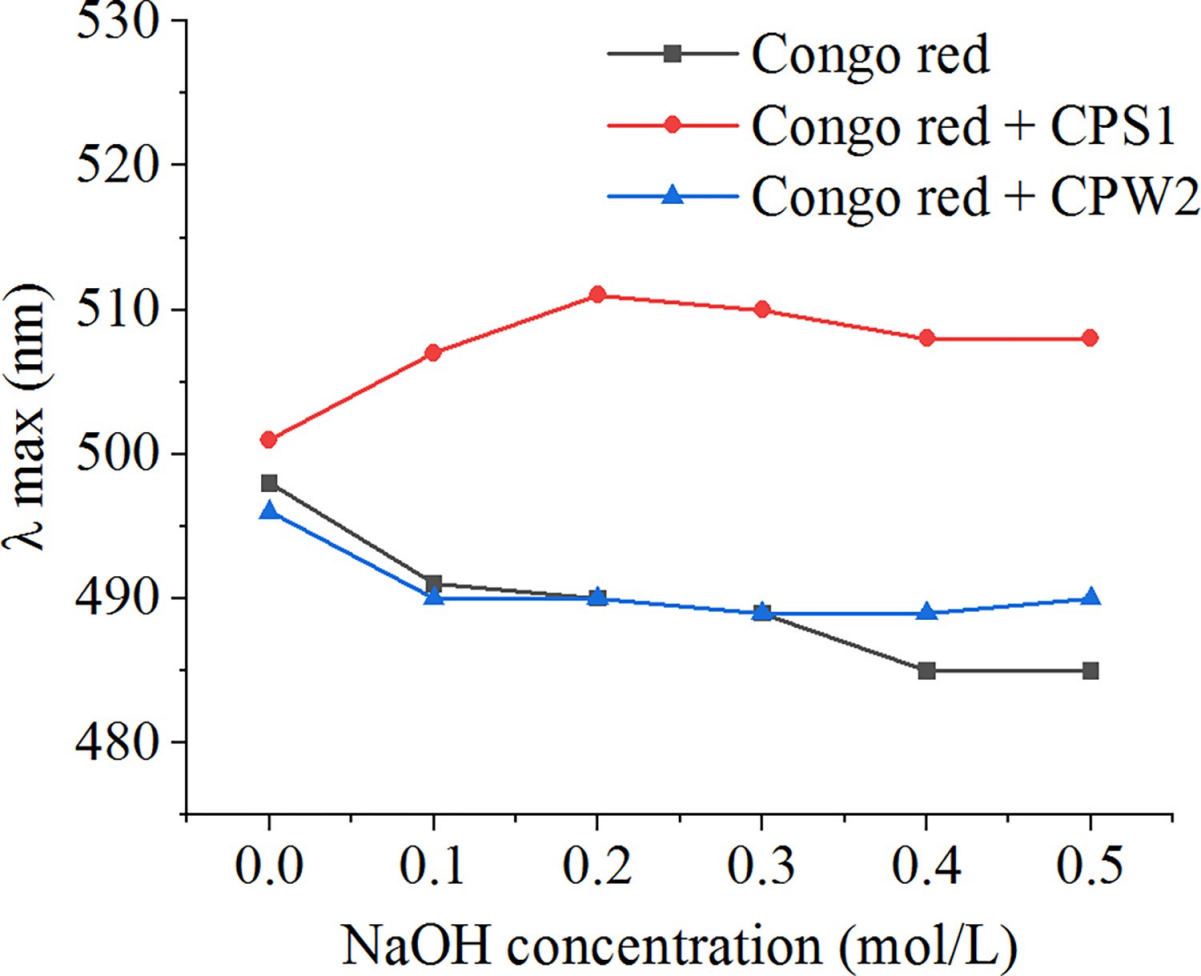
The maximum absorption wavelengths of Congo red-polysaccharide (CPS1 or CPW2) complex at various concentrations of NaOH.

Congo red tests of CPS1 and CPW2 were conducted and the results were presented in Fig 6. Compared with the Congo red control, λ_max_ values of Congo red-CPS1 complexes showed large redshifts and that of Congo red-CPW2 complexes had a similar trend, suggesting that CPS1 molecules possessed helix conformation and CPW2 had no that [20]. For CPS1 molecules, λ_max_ values decreased gradually under stronger NaOH conditions (more than 0.2 mol/L), and a decline in redshift effects was observed, which was due to the destruction of the helix structure in CPS1 molecules. The results from Congo red tests agreed well with the above SEM and TEM analyses.

### Anti-inflammation effects

The inhibitory effects of CPS1 and CPW2 for cell proliferation were analyzed using the MTT method, and the dosages of CPS1 and CPW2 in the following anti-inflammation tests were determined to be 75 and 150 μg/mL final treatment concentrations which did not influence the proliferation of RAW 264.7 mouse macrophage cells.

**Fig 7:**
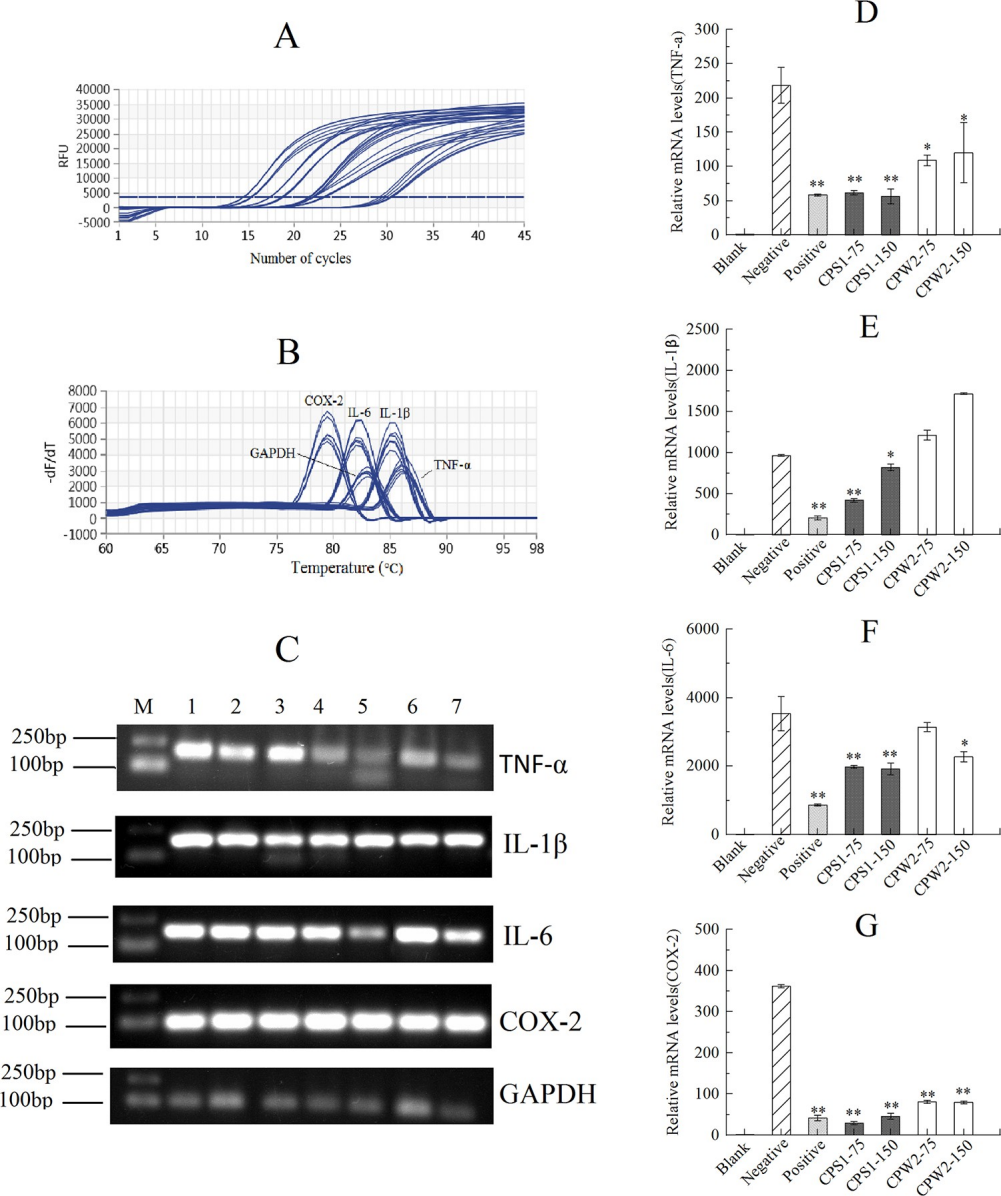
Amplification. (A) and melting peak curves (B) of genes for CPS1 and CPW2. Electrophoresis diagrams of amplified genes (C) (M: D15000+2000 Marker, 1–7: Blank, negative, positive, CPS1-75, CPS1-150, CPW2-75, and CPW2-150 groups, respectively). TNF-α (D), IL-1β (E), IL-6 (F) and COX-2 (G) mRNA expressions in LPS-stimulated RAW264.7 mouse macrophage cells (compared with the negative control, * *P* < 0.05; ** *P* < 0.0001). Each data point represents the mean ± SD (*n* = 3).

Relevant inflammation factors including TNF-α, IL-6, IL-1β, and COX-2 were used to evaluate the anti-inflammatory effects of CPS1 and CPW2 molecules in LPS-induced RAW 264.7 cells, and the results from real-time PCR tests were demonstrated in Fig 7A and 7B. Amplification curves of GAPDH, TNF-α, IL-6, IL-1β, and COX-2 genes showed to be S-shaped curves, and melting peak curves presented a single peak for each gene (marked as gene names in Fig 7B), which indicated the validity of real-time PCR tests and the good primer specificity for the amplified genes. Electrophoresis diagrams of amplified genes were shown in Fig 7C, where all the molecular weights for the amplified region of each gene were between 100 bp and 250 bp, which was consistent with the expected results.

According to the 2^-ΔΔCT^ method, the suppression effects of CPS1 and CPW2 on TNF-α, IL-6, IL-1β, and COX-2 mRNA expression in LPS-induced RAW 264.7 cells were estimated and the results were shown in Fig 7D–7G. By comparison of data with blank groups, all of the mRNA expression levels in negative groups were significantly increased by LPS (*P* < 0.0001), which indicated that all inflammation models had been successfully constructed. The inhibitory effects of CPS1 and CPW2 on LPS-induced mRNA expressions of inflammation factors were obtained based on the comparison of data from a sample and negative groups, and the results were summarized in Table 2, where the suppression effects of CPS1 on the TNF-α, IL-1β, IL-6, and COX-2 mRNA expressions were 71.80% (*P* < 0.0001), 56.55% (*P* < 0.0001), 43.98% (*P* < 0.0001) and 91.88% (*P* < 0.0001) at the 75 μg/mL final concentration, respectively. The CPW2 molecule at the 75 μg/mL final concentration was also able to suppress mRNA expressions of TNF-α (50.07%, *P* < 0.05), IL-6 (11.07%, ns) and COX-2 (77.55%, *P* < 0.0001), while CPW2 showed lower inhibitory effects in the test concentrations compared to CPS1 molecules.

**Table 2:** Inhibition effects of CPS1 of CPW2 on the expression of inflammatory factors in LPS-stimulated RAW264.7 cells.

Factors	Inhibition ratios(%)			
	CPS1-75	CPS1-150	CPW2-75	CPW2-150
TNF-α	71.80	74.22	50.07	45.07
IL-1β	56.55	14.98	-^a^	-^a^
IL-6	43.98	45.83	11.07	35.55
COX-2	91.88	87.31	77.55	78.03

^a^ Not detected.

In addition, at the experiment concentrations, the inhibitory effects of CPS1 on TNF-α and COX-2 mRNA expressions had no significant difference (*P* > 0.05) by comparing with positive groups. These results showed that CPS1 with the four-helix structure possessed more significant anti-inflammation effects than CPW2 without entangled chains.

### Antioxidant activities

**Fig 8:**
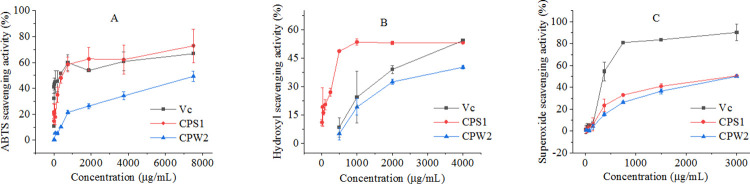
Scavenging activities for ABTS (A), hydroxyl (B), and superoxide (C) radicals of CPS1, CPW2, and Vc.

In the present study, different methods including superoxide, hydroxyl, and ABTS radical scavenging assays were used to estimate the antioxidant activities of CPS1 and CPW2, and the results from antioxidant tests were presented in Fig 8. Vitamin C (Vc), as the positive control, was assigned. As shown in Fig 8A–8C, the scavenging activities of CPS1 and CPW2 for all the radicals including superoxide, hydroxyl and ABTS were dose-dependent.

For ABTS radicals, CPS1 showed a good scavenging capacity while exhibiting a trend similarity to Vc as demonstrated in Fig 8A. In detail, 72.91% of ABTS radicals could be inhibited by CPS1 at 7500 μg/mL treatment concentration and the scavenging activity of CPW2 at the same dosage was 49.25%, by which we could conclude that CPS1 had a stronger ABTS radical scavenging ability than CPW2. According to the scavenging activities, the EC_50_ value of CPS1 for the ABTS radical scavenging ability was calculated to be 533.99 μg/mL. The hydroxyl radical scavenging capacity of CPS1 was more than 50% at the final concentration of 1000 μg/mL, while the scavenging activity of CPW2 at the same dosage was less than 25%, and then the EC_50_ value of CPS1 for the hydroxyl radical scavenging effect was determined to be 520.46 μg/mL. Based on the scavenging tests for superoxide radicals, the EC_50_ value of CPS1 was calculated as 1512.06 μg/mL, which was better than that of CPW2 (less than 50% of scavenging activities at all the treatment concentrations). In addition, CPS1 molecules (EC_50_ = 520.46 μg/mL) exhibited a stronger hydroxyl radical scavenging ability than Vc (EC_50_ = 2513.06 μg/mL, *P* < 0.01).

In summary, CPS1 with the four-helix structure showed more excellent antioxidant activities than CPW2, and it even had a more outstanding performance in terms of hydroxyl radical scavenging ability compared to Vc. Hydroxyl radicals, normally termed the most active radicals, may cause serious damage to biomolecules in cells and give rise to cytotoxicity and cancer [33].

## Conclusions

In this study, optimal extraction conditions of total sugars from *C*. *decumbens* were obtained. Two polysaccharides (CPS1 and CPW2) were isolated and purified from *C*. *decumbens*, and they possessed different structural characteristics and bioactivities. The Mw of CPS1 was 360 kDa, and the CPS1 molecule possessed a four-helix conformation with side chains and comprised glucose, galactose, mannose, and arabinose. The CPW2 molecule was composed of glucose with 550 kDa Mw, and the molecular microstructure displayed linear chains without branched and entangled conformations. In the term of anti-inflammatory activity, CPS1 exhibited more outstanding suppression effects on TNF-α, IL-1β, IL-6, and COX-2 mRNA expressions in LPS-stimulated RAW 264.7 cells than CPW2 (*P* < 0.0001), in which the inhibitory activities of CPS1 for TNF-α and COX-2 mRNA expressions even had no significant difference with the positive group (*P* > 0.05). The more excellent scavenging abilities for hydroxyl and ABTS radicals were also evidenced in CPS1 than CPW2, and then CPS1 (EC_50_ = 520.46 μg/mL) showed a stronger capacity of scavenging hydroxyl radical compared to Vc (EC_50_ = 2513.06 μg/mL).

For polysaccharides, bioactivities are likely to be influenced by their structural features such as Mw, monosaccharide compositions, and chain conformations [34,35]. The chain conformation might play the most important role among structural features and it has been reported that the triple-helical conformation in polysaccharide molecules may be related to outstanding bioactivities [32]. However, there are no reports concerning polysaccharides from *C*. *decumbens* and the four-helical structure of polysaccharides. CPS1 possessed the four-helical structure, which may be contributed to its good bioactivities. These results provide new information on the four-helical structure concerning higher anti-inflammatory and antioxidant attributes and give insights into the potential application of polysaccharides from *C*. *decumbens* in the field of medicine.

## Supporting information

S1 Fig Original gel images of amplified genes TNF-α, IL-1β, IL-6, COX-2, and GAPDH with the marker of D15000+2000. (TIF)
