# Supplementary figures and images for: Extraction optimization, structure features, and bioactivities of two polysaccharides from Corydalis decumbens
Source: PLoS One. 2023 Apr 13;18(4):e0284413. doi: 10.1371/journal.pone.0284413 (PMC10101462; doi:10.1371/journal.pone.0284413)

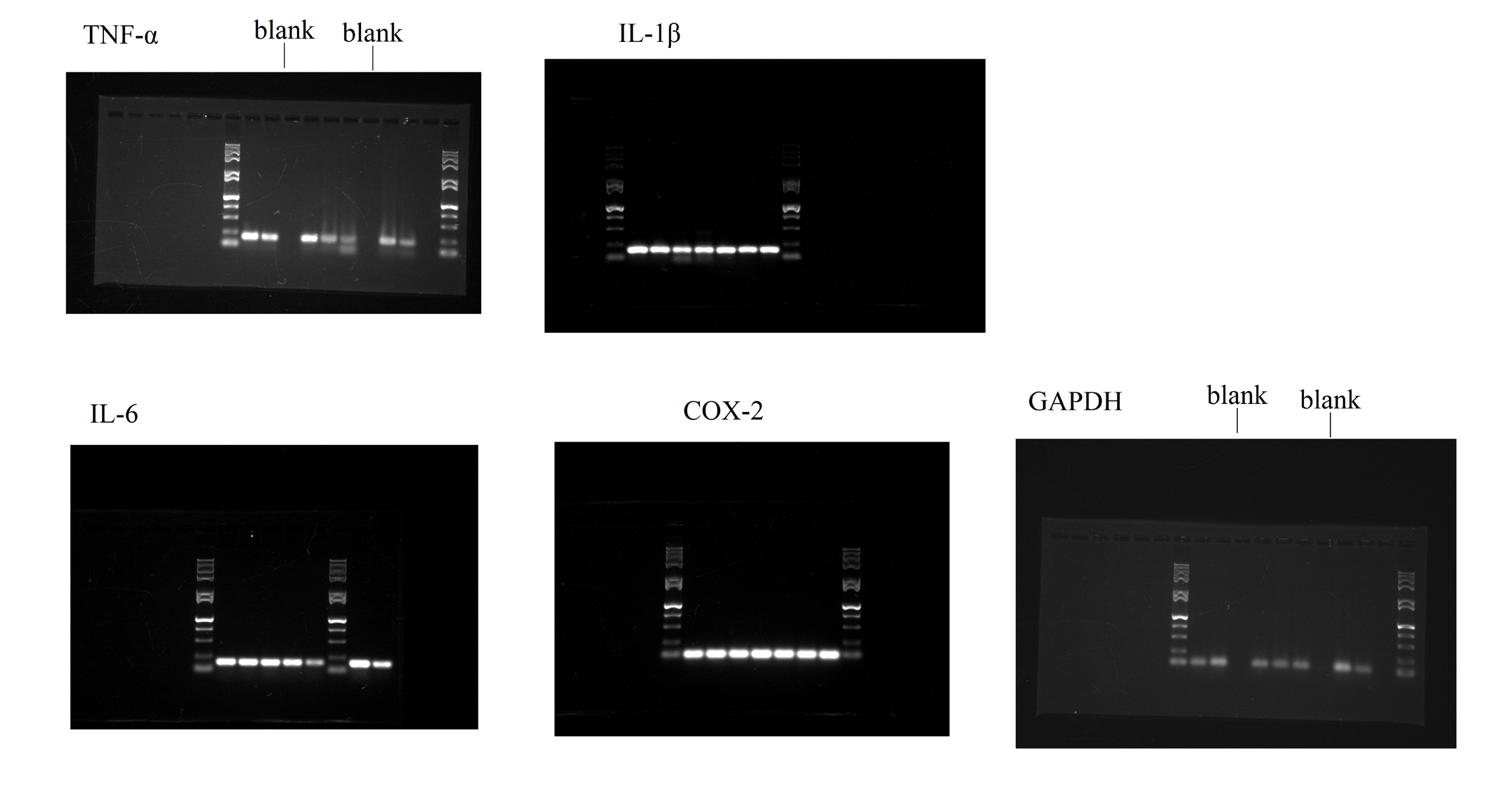

Supplement: S1 Fig — (TIF) [file pone.0284413.s001.tif]
